# Relationship between Addiction Relapse and Self-Efficacy Rates in Injection Drug Users Referred to Maintenance Therapy Center of Sari, 1391

**DOI:** 10.5539/gjhs.v6n3p138

**Published:** 2014-02-28

**Authors:** Zahra Abdollahi, Fatemeh Taghizadeh, Zeinab Hamzehgardeshi, Olia Bahramzad

**Affiliations:** 1 Department of Midwifery Education, Nasibeh Nursing and Midwifery Faculty, Mazandaran University of Medical sciences, Sari, Iran; 2 Department of Mental Health, Public Health Center, Mazandaran University of Medical sciences, Sari, Iran; 3 Traditional and Complementary Medicine Research Centre, Mazandaran University of Medical Sciences, Sari, Iran; 4 Department of Midwifery, Mazandaran University of Medical Sciences, Sari, Iran; 5 Department of Midwifery, Public Health Center, Mazandaran University of Medical Sciences, Sari, Iran

**Keywords:** self-efficacy, relapse, addiction, methadone maintenance therapy center (MMTC(

## Abstract

**Background and Purpose:**

Self-efficacy is the belief that one has the ability to implement the behaviors needed to produce a desired effect. There has been growing interest in the role of self-efficacy as a predictor and/or mediator of treatment outcome in number of domains. In numerous studies of substance abuse treatment, self-efficacy has emerged as an important predictor of outcome, or as a mediator of treatment effects. In the event of a slip, highly self-efficacious persons are inclined to regard the slip as a temporary setback and to reinstate control, whereas those who have low self-efficacy are more likely to proceed to a full-blown relapse. This study was carried out to determine relationship between relapse and self-efficacy and other factors in injected drug users.

**Materials and Methods:**

We conducted this study in 200 addicts in the center of counseling behavioral disease in health center of sari city (methadone maintenance therapy center or MMTC). A cross-sectional study was carried out on all of these addicts.

**Results:**

The average age in addictions was38 and its range was 20-60.72%of them were married and the first drug used was opium. All of them had relapse at least one time. We found a relationship between relapse and self-efficacy as well as the relationship between self-efficacy with the age of the first of drug use, dose, and procrastination for treatment, marriage, employment and job was significant.

**Conclusion:**

This study found that there was a significant difference between relapse and self-efficacy as well as other related factors. It is important to include drug users and common society organizations representing them in every stage of the governmental policy and program development process to make them responsive to the needs of the community.

## 1. Introduction

Drug abuse and addiction is a major global problem that destroys economy health, relationships and career and has several complications including relapse that often remain untreated (Nessa, Latif, Siddiqui, Hussain, & Hossain, 2008). Substance use has chronic relapsing period (Gaily & Bashir, 2004). The literature review showed that the self-efficacy related to addiction relapse (Ibrahim, Kumar, & Samah, 2011). In addition to the treatment addict person who have high self-efficacy would be low risk to be substance user again (alsop2000). The past studies showed that there is strong relationship between self-efficacy with addiction relapse (Dolan, Martin, & Rohsenow, 2008; Ibrahim, et al.,2011). One of the most important factors that influence the rate of relapse is not enough self-efficacy in drug addicts. Self-efficacy is derived from social cognitive theory of famous psychologist that refers to beliefs or judgments of individual about his capabilities to perform the duties and responsibilities. Social cognitive theory is based on a tripartite model of the environment and the individual. Social cognitive theory is based on a tripartite model of the behavior, environment and the individual. This model emphasized the relationship between behavior, environmental effects and personal factors (cognitive, emotional and biological factors) which refers to perception of psychological functions. According to this theory, people in a tripartite causal system affect their behavior and motivation (Bandura, 1999). Bandura has rejected environmental effects on individual behavior that is one of behavioral psychologists’ assumptions. Humans have a system of self-regulation and self-control and by this control his thought, feelings and behavior and also have decisive role on your own destiny. Thus, human behavior is not only control by environment but also cognitive processes have an important role in human behavior. Human learning and performance affected by cognitive approaches, emotional, expectations, beliefs and values. Human being is an active creature and affects his life events. Human is influenced by psychological factors and actively affect his motivation and behavior. According to “Bandura” People are not driven by internal forces, nor do environmental stimuli lead them to action, thus psychological functions determine function, behavior, environment and their stimulations. Bandura argues that self-efficacy, which thereby can be constructive, cognitive skills, social, emotional and behavior for different goals, such regulation is effective. In his idea having knowledge, skills and previous achievements are not good predictor of future performance, but people believe about their abilities to perform their functions are effective. There are significant differences between varieties of skills with their combination power in appropriate methods and in different conditions. “People know what to do and have skills needed to perform their functions, but often fail to implement appropriate skills” self- recognition enabled through cognitive, motivational and emotional process which are responsible for transfer of knowledge and abilities to conduct. In summary, self-efficacy is not related to a skill or skills, but also refers to belief in ability to work in different job situations. Self-efficacy theory predicts that treatment will be effective when increase the client’s reasonable expectations of what can be achieved and continue (Bandura, 1999). Sense of self-efficacy is an individual’s judgment about their ability to perform an action that can enable people to adopt healthy behaviors and leave harmful ones (Bandura, 1999). A study from Iran investigated the relationship between confidence and self-efficacy with health behaviors of Yazd students with higher self-efficacy in medical university students their health scores increased. Significant positive correlation between health behavior and self-confident students were observed (Mazloumi Mahmoudabad, Mehri, & Morovati Sharifabad, 2006). Self-efficacy of a treatment increases the need to a business and health promotion behaviors; behavior change is the most important factor (Bandura, 1999). Since addiction is an extremely stressful behavior and their role in its effectiveness is obvious; independent research so far in relation to addiction relapse rate of drug efficacy have not looked in Mazandaran province so we decided to do the study. This study was a cross - sectional study in which the researcher examines the relationship between addiction relapse and self-efficacy and its relationship with some factors in injection drug users referred to the health center of Sari city. The study population consists of all drug users of treatment center in Sari. Samples consist of all drug users in Sari Health Maintenance Treatment Center. Samples were selected through census sampling, in this study 200 drug users in were examined in Sari health maintenance treatment center. This is a cross - sectional study that is done on all injection drug users referred to the state health department at Sari- Iran (about 200 people by census). First, interviewers were trained. Then the purpose of self-administered and self-efficacy questionnaire design and methods has been described for drug addicts and the questionnaire was completed by interview and questionnaire was completed by addicts.

## 2. Methods

In this study, three types of questionnaires were used as follows: firstly, Epidemiology of subjects; secondly, The questionnaire related to drug abuse type, history of addiction, Addiction Withdrawal decision, withdrawal action and relapse; and thirdly; Standard questionnaire for measuring efficacy.

For checking reliability of questionnaire internal consistency was used and reduced Cronbach’s alpha was measured 0/88.

General Self-Efficacy Scale consists of ten questions that have been used in several investigations in Iran in this study score above 35 indicate high efficacy and below 30 low efficacies and the rest are average. Grading scores is done by Likert method (1-4). The findings based on the type of data, quantitative continuous (age) discrete quantitative, such as frequency of use, qualitative data such as the level of education and occupation. Following data, descriptive and inferential statistics were used to analyze the data.

Descriptive statistics are used as frequency distribution tables, mean and standard deviation. In inferential statistics SPSS version 16 software was used. Descriptive and analytical indicators such as Pearson coefficient were determined. The chi-square test was employed for the bivariate analysis. The level of significance was set at 0.05.

## 3. Results

In this study, mean age is 38 years old and its domain is between 20-60 years. 28% of participants were single and 72% are married and 74 percent of them have self-employment, 21% of them are unemployed and 5% are retired. In terms of education, 43% were illiterate or have primary education, 57 percent junior school, 71 percent have diploma, 20 percent associate degree and 9 percent have bachelor degree or higher. According to Table 2-4 their father education level in 67 percent is illiterate and primary level education, and also 66 percent of their mothers are illiterate and primitive. The first intake 63 percent were opium, in 32 percent were Hashish and in 5 percent were alcohol; the way of using drugs in 76 percent was smoke, in 19 percent oral and in 5 percent inhalation. The reason of relapse after withdrawal in 53% of subjects was temptation, in 15% friends and in 17% emotional and family problems. The mean age of first drug use was 19 years, and the frequency of 2 times per day. Decision to quit the drug takes average 4 years after the first intake, after decision to stop it takes 32 months to start treatment with methadone. After first withdrawal averagely takes 7 months that people reuse the drug. Addicts averagely have had 8 withdrawals and interval between their withdrawals was 6 months. 58% of them used all materials, and the majority of them 56 percent used. In 58 percent one of the family members use drugs, in 18% father, in 21 percent brother, in 8% wife, in 5% son and in 7% all family members. 40% of people at least once have been admitted to a hospital or treatment center. 67% have been tested for HIV and 26 percent had sex, at least once, with a strange person. According to Table 4.10, the mean of self-efficacy in this study is 5/32 and its domain is between 24-40 that from this amount 45 percent have low self-efficacy <30, 25 % have moderate self-efficacy (30-35) and 30% have low self-efficacy (scores above 35). Other findings are as follows: there is a significant relationship between withdrawal times and self-efficacy (p<0.05). However, there is no relationship between frequency of use and the age of first use, but there is a direct relationship between withdrawal and self-efficacy (p<0.05). Between withdrawal and first relapse (clean time) self-efficacy according to this point that the amount of p-value is less than 0/05, this result is obtained that there is a direct and significant relationship between two variables. There is a significant and inverse relationship between the visit times from hospital due to drug overdose and self-efficacy. Also, self-efficacy in married is more than singles and this relationship is significant and direct (p<0.05). Self-efficacy in people that their father has higher education is more (p<0.05). Efficacy in people which their mother have higher education is more (p<0.05). Individuals that their father is unemployed have lowest self-efficacy score and those who their fathers are self-employed have highest self-efficacy.

**Table 1:** Distribution of respondents according to the first drug, and how to use and re-use after withdrawal

Drug	Frequency	Percent
Hashish	64	32%
opium	126	63%
Alcohol	10	5%
Total	200	100%
Way of use	Frequency	Percent
Smoking	152	76%
Inhalation	10	5%
Edible	38	19%
Total	200	100%
The reason for re-use	Frequency	Percent
Temptation	107	53%
Emotional problems	14	7%
Family problems	23	10%
Friends	30	15%
Without answer	30	15%
Total	200	100%

**Table 2:** Table of variables in terms of the mean

Variables	Mean
How old were you the first time you use?	19
How many times did you use?	3
The first time after how many years you’ve decided to leave?	7
After deciding to leave it took how many months to begin methadone treatment?	32
After first withdrawal it took how many months to use again?	7
How many times have you leaved?	8
How many months gap are there between your withdrawals?	6

**Table 3:** Table of some variables related to self-efficacy

Row	Variables	Pearson coefficient	Error rate α	p.va	Significance
1	Age of the first consumption with self-efficacy	-0/058	0/05	0/4	Not significant
2	The number of daily consumption with self-efficacy	-0/01	0/05	0/9	Not significant
3	Distance between withdrawal and first relapse (clean time) with self-efficacy	0/2	0/05	0/005	Significant and positive
4	Delay in treatment with self-efficacy	-0/28	0/05	0/001	Significant and reversed
5	Frequency of withdrawal with self-efficacy	0/19	0/05	0/02	Significant and positive

## 4. Discussion and Conclusions

In this research samples include 27% singles and 72% married. Also 74% of them have free job and 21% are unemployed and 5% are pensioners. Also in an Iranian research 12% of mans are unemployed (Seraji, Momeni, & Salehi, 2010). Base on the table of sample in term of education, 43% illiterate and primary school, 57% junior, 71% diploma, 20% associate degree and 9% bachelor degree and higher. Fathers education 67% on illiterate and primary school level and also 66% of mothers are on illiterate and primary school level. Age average of this research is 38 and its amplitude is between 20 and 60.

First used drugs is opium for 63% of mans, Marijuana for 32% of mans and alcohol for 5% of them. Also using method was smoking for 76% of addicts, meal for 19% and inhalation for 5% (Table 1). Opium was the most common used drug in Ahari and associates research (Narimani & Sadeghieh, 2008).

Table 3-4 show that Cause of relapse after quitting was temptation for 53% of samples, 15% friends and 17% sentimentally and domestically problems. Most important environmental cause of addiction relapse discussed respectively, sleepless and temptation, psychological distress, Deficiency of confidence and feelings of futility and ramble in the Iranian associates research (Mirzaei et al.,2011). In a study psychological parameter such as Anxiety, stress, depression, feelings of losing something, availability of drug’s, socializing with addict friends, belief that they will not addict by once drug use and self-examination were the most common factors of addiction relapse in turkey (Narimani & Sadeghieh, 2008). Age average of first drugs usage is 19 years old and the number of iterations per day was 2 uses per day. Deciding to quit the drug mean lasted 4 years after first use. Samples also after decide for quitting lasted on mean 32 month until starting treatment by methadone. After first drug quitting lasted on mean 7 month to relapse addiction. Based on past study, 53% of addicts relapse addict less than 3 month and just 12% of them could be stay without drugs more than 1 year and average of quitting was 6.3 month (Mirzaei et al.,2011). In another study 72% of mans had relapse (Narimani & Sadeghieh, 2008).

Addicts on mean have 8 times quitting and average time between their addict quitting was 6 month and 58% of them used all of drugs and majority namely 56% used crystal. A study reported that 35% of relapses occur in the negative emotions, 16% in conflicts with others, and 20% for social pressures (Marlatt & Donovan, 2005). Another study concluded that 62 –73% of relapse episodes coded under negative emotions and social pressures (Lowman, Allen, & Stout, 1996).

Previous study found that social pressures determinants were not the only important factors in relapse, but craving, temptation, and substance cues can also do so (Bradley, Phillips, Green, & Gossop, 1989). Some research showed that heroin addicts relapse primarily because of NE and lack of social supports. Mood state, along with social isolation and family factors, was more likely to be repeated as high-risk situations of the coming relapse incidences (Heather, Stallard, & Tebbutt, 1991).

In a Persian study, 33% of addicts once, 38% 2-3 time, and 28.5% of them more than tree time have unsuccessful treatment (Mirzaei et al.,2011). In addition, another study reported 48.9% of mans have addiction relapse within first 4 month after quitting (Seraji et al.,2010). Also there isn’t relation between use frequency and so first use age with self-efficacy. But between drug quitting time and self-efficacy there is direct and significant relation (p<0.05) depend on p value amount less than 0.05 between quitting and firs relapse (clear time) that concluded between two variant there is significant relation and it’s direct. This point show that mans with higher self-efficacy has more time strength against relapse than addicts with low rate self-efficacy. There is significant and reverse relation between counts of refers to the hospital because of drugs over dosage and self-efficacy. Also married have more self-efficacy than single mans and this is a direct and significant relation (p<0.05). A number of relatively recent studies assessing the role of self-efficacy among abusers of various substances are cited, but the list is not meant to be exhaustive (Kadden & Litt, 2011).

Many studies have shown that self-efficacy is a predictor of treatment outcome. In some cases, self-efficacy has been found to predict the quantity of alcohol or drugs consumed. These studies found that self-efficacy significantly predicted alcohol consumption for periods of up to twelve months also argued that negative life events and the exposure to the high-risk situation had not been related to relapses probability (Maisto, Connors, & Zywiak, 2000). However, another study found that higher self-efficacy predicted less drug use only after 3 months but not after 6 months (Dolan et al.,2008). In a study of the Effectiveness of step-down continuing care following residential or intensive outpatient care found little evidence to support step-down continuing care itself (McKay et al.,2004). Other study have shown that self-efficacy was a relatively strong predictor of post-treatment abstinence and the frequency of marijuana use (Kadden & Litt, 2011) also reported a significant relationship between self-efficacy expectancies during inpatient alcohol dependence treatment and several frequency-related outcome variables:The likelihood of drinking; time to first drink; and time to relapse during the year following treatment similarly for outpatient treatment. Alcoholics’ post-treatment self-efficacy was a predictor of time to relapse and high confidence in their ability to resist drinking were more likely to maintain abstinence for 6 months (Borrelli & Mermelstein, 1994). A study observed that individuals whose increased confidence in high-risk situations persisted during follow-up had both fewer days of use and reduced alcohol/drug severity (Brown, Seraganian, Tremblay, & Annis, 2002).

The past study reported a negative relationship between self-efficacy and relapse to alcohol use, but not for relapse to drug use (Walton, Blow, Bingham, & Chermack, 2003). In a study comparing four treatment approaches for marijuana dependence (Romo et al.,2009), while replicating the common finding that high self-efficacy was correlated with longer periods of abstinence.

Given the low level of self-efficacy in this study and its relationship with relapse, specifies the need for interventions to increase these variables in addicts.

Consistent with other studies, the results suggest that self-efficacy factor is an important factor towards relapsed addiction amongst addicts (Ibrahim et al.,2011). With efforts to enhance the preparation of total human development strategy amongst relapsed drug addicts, it could increase addicts’ self-efficacy to live without drugs. It means that serious efforts should be done to restructure weak self-efficacy to enable the addicts to be stronger when facing life challenges after their release. Although the findings of this study are important for the stakeholders in public health, it is also essential to conduct future studies with larger sample sizes.
